# Adherence to Palivizumab for Respiratory Syncytial Virus Prophylaxis in Romanian Infants

**DOI:** 10.3390/vaccines13020171

**Published:** 2025-02-10

**Authors:** Andreea Calomfirescu-Avramescu, Adrian Ioan Toma, Claudia Mehedințu, Leonard Năstase, Vlad Dima

**Affiliations:** 1 Filantropia Clinical Hospital, 011171 Bucharest, Romania; 2 Faculty of Medicine, Titu Maiorescu University, 040441 Bucharest, Romania; 3 Obstetrics-Gynecology and Neonatology Department, Carol Davila University of Medicine and Pharmacy, 020956 Bucharest, Romania

**Keywords:** respiratory syncytial virus, immunoprophylaxis for RSV, palivizumab

## Abstract

Background: In 2022, Romania started an RSV immunoprophylaxis program with Palivizumab for infants at high risk: preterm infants born before 35 weeks of pregnancy, infants born with congenital heart defects, and infants with chronic lung disease. We evaluated treatment adherence from August 2022 to March 2024. Method: We monitored the increase in the number of patients enrolled in the program and the number of collaborating neonatologists, family doctors, and pediatricians. Adherence to all doses of Palivizumab in enrolled patients was assessed by telephone interviews. The factors contributing to reduced adherence were identified. Results: Between August 2022 and March 2024, 1903 patients and 233 specialists were enrolled, a steady increase in both cohorts. The percentage of patients that complete their full sequence of doses decreases along with the number of doses (99% for one dose, 73% for two doses, 47% for three doses, 35% for four doses, and 22% for five doses) due to several factors. Conclusions: The program remains highly regarded by both physicians and caregivers, demonstrating its effectiveness as a valuable resource for educating parents and facilitating monoclonal antibody administration as a prevention method for RSV.

## 1. Introduction

Respiratory syncytial virus (RSV) is linked to considerable mortality and morbidity in pediatric populations. Although there is significant progress in preventing, diagnosing, and treating RSV infections, this virus remains the predominant cause of lower respiratory tract infections in infants under one year of age [1].

### 1.1. Epidemiology

A 2017 meta-analysis revealed that the global annual rate of RSV hospitalization in children under 5 years was 4.4 per 1000, with the highest rates occurring in infants under 6 months and preterm neonates [2]. Another meta-analysis indicates that RSV accounts for 2.3% of global mortality in neonates under 28 days, 6.7% in infants aged 28–364 days, and 1.6% in children aged 1–4 years [3]. Furthermore, according to a 2022 systematic research, RSV causes 3.6% of deaths in children ages 28 days to 6 months and 2% of mortality in children ages 0 to 60 months. Low-income and middle-income countries (LMICs) accounted for over 95% of RSV-associated acute lower respiratory infection episodes and over 97% of RSV-attributable deaths across all age groups [4].

RSV infection incurs elevated healthcare expenses, as the vulnerable populations (premature infants under 35 weeks of gestation, individuals with chronic lung disease, and those with heart abnormalities) necessitate several hospital admissions [5]. A 2016 study revealed considerable cost variations across hospitalization categories: term infants with hospitalized RSV infection incurred USD 9014; newborns with a gestational age under 33 weeks incurred USD 13,876; and newborns with a gestational age between 33 and 36 weeks incurred USD 18,403 [6].

The virus is transmitted through nasal or oral secretions and persists in the blood between 3 and 8 days. It can persist longer in immunocompromised people, especially in HIV-positive children [7]. The incubation period is between 2 and 8 days [8]. Recurrent infections are commonly observed.

RSV infection exhibits seasonal variations, differing by country. In the Northern Hemisphere, RSV seasonal epidemics transpire from October to April; in contrast, in the Southern Hemisphere, they occur from May to September [9,10]. Additionally, in tropical countries, seasonal illnesses generally vary during the rainy season. Epidemic peaks are relatively less severe in temperate regions compared to tropical and subtropical climates. The RSV season in Romania occurs from October to March. Furthermore, during the COVID-19 epidemic, mitigating measures resulted in a reduction in infection rates [11,12,13,14].

Respiratory syncytial virus infection predominantly occurs in children under 24 months, with an incidence of 5.2 per 1000 (26 per 1000 in neonates within the first month post-birth). Hospitalization rates increase within the initial six months [15]. The infection is more common in babies because their IgG levels are reduced, reaching a nadir between 3 and 6 months. Especially in preterm infants, whose immune systems are immature and immunoglobulin G levels are markedly diminished.

Patients at the highest risk include the following:

- Infants and children with chronic lung disease (bronchopulmonary dysplasia, cystic fibrosis) [16];
- Preterm infants are less than 35 weeks gestational age [17];
- Infants with congenital heart disease [18];
- Infants exposed to second-hand smoke [19];
- Immunocompromised patients (HIV) [20];
- Infants with genetic disease (Down syndrome) [21].

During their research, Shi and colleagues developed a Risk Scoring Tool (RST) for RSV infection. This tool considers the following factors: birth during the RSV epidemic time, maternal smoking, as well as smoking in the home, exposure to possible infected individuals with RSV (sibling, daycare attendance), and so on. We frequently use this score to determine the viability of RSV prophylaxis [22,23]. Additionally, the gestational age, chronological age at the beginning of the RSV season, birth weight, and male gender are all considered to be significant risk factors for RSV infection that necessitate hospitalization.

Researchers have established a statistically significant correlation between risk score and risk categories, and they have verified the RST both nationally and globally. Researchers from Spain (FLIP-2, Spain) [24], The Netherlands (RISK) [25], Canada (PICNIC, Canada) [26], Italy (Italian National Birth Cohort) [27], the USA (REPORT, USA) [28], and the U.K. (PONI, UK) [29] used studies from their countries to back up their claims. The Spanish study examined risk variables associated with respiratory syncytial virus infections necessitating hospitalization, particularly in premature newborns; the Dutch study investigated the at-risk group, while the Canadian study focused on pediatric investigators.

The scoring system spans from 0 to 56, categorizing scores of ≤19 as low, 20–45 as moderate, and ≥50 as high risk. The hospitalization rate in the high-risk group was 9.5%, whereas in the extremely high-risk group, it was 11.9% [26]. The IMpact randomized controlled study demonstrated that administering Palivizumab as a prophylactic intervention reduced RSV hospitalizations by 80–85% in infants delivered between 32 and 35 weeks of gestation. The required number for treatment was 12 [30].

RST is an effective tool for identifying children in need of prophylaxis, especially given the constrained financial means for universal provision. A 2018 study revealed that merely 16% of hospitalizations resulting from respiratory infections attributed to RSV were administered preventive medicine [31].

### 1.2. Pathophysiology

Following its entry into the body, the respiratory syncytial virus travels to the respiratory system, where it attaches itself to the apical ciliated epithelial cells. Inflammation and necrosis occur in the respiratory system because of its presence [32]. The immunological response is mediated by IL-8, and the severity of the disease is significantly associated with the immune response. Previous RSV infections do not offer immunity against subsequent infections [33].

More severe cases may involve alveolar blockage. Furthermore, there are further downstream effects, including ciliary dysfunction, resulting in compromised mucus clearance, airway edema, and reduced lung compliance.

Humoral immunity mitigates the severity of RSV infections, resulting in less severe recurring infections. Elevated transplacental RSV antibody titters correlate with less severe symptoms confined to the upper respiratory tract. Reduced antibody titters in cord blood correlate with a heightened likelihood of RSV hospitalization before 6 months of age. RSV ascends from the nasopharynx to the small bronchiolar epithelium, then advancing to type 1 and 2 alveolar pneumocytes [34,35].

### 1.3. Clinical Presentations

The age of the child when the illness first manifests and the clinical signs that are present are correlated. Newborns most commonly experience three symptoms: fever, cough, and coryza. Between 1.2 and 23.8% of cases can be linked to apnea [36]. Symptoms such as rhinitis, pharyngitis, tachypnea, otitis, wheezing, and conjunctivitis can be seen in infants between the ages of six weeks and six months. This infection can take place on its own or in conjunction with other diseases, depending on the circumstances [37].

Bronchiolitis, bronchospasm, and pneumonia are all symptoms that can be brought on by severe respiratory syncytial virus infections [38]. The clinical severity of the original infection is the highest, and the severity of subsequent reinfection episodes increases with each successive infection [39]. There is a correlation between severe cases that require assisted ventilation and certain outcomes, one of which is hyponatremia caused by the improper release of antidiuretic hormone [40].

### 1.4. Diagnosis

Clinical signs of bronchiolitis serve as a basis for guiding the diagnostic process. Laboratory diagnosis is necessary for severe or unusual cases or to ascertain the need for Palivizumab prophylaxis [41].

Blood inflammatory tests show a slight rise in C-reactive protein (CRP), although they are not specific. Testing for RSV is not recommended unless its detection would influence medical decision-making. Targeted testing for RSV can aid in distinguishing it from other conditions and is offered in two prevalent modalities: fast antigen testing and polymerase chain reaction (PCR) testing. Rapid antigen diagnostic tests (RADT) yield results within 30 min. A meta-analysis of 71 studies indicates that the sensitivity in children is 80% and the specificity is 97%. Retesting may be required for patients with false-negative results [42]. The RADT can be utilized for screening, and negative results may be validated by PCR [43]. It is less sensitive than PCR-based assays [43]. Palivizumab prophylaxis may interfere with RADT, leading to false-negative results [41].

Reverse transcriptase polymerase chain reaction (RT-PCR) provides rapid, dependable results with enhanced sensitivity relative to culture and rapid antigen assays and is unaffected by passively supplied antibodies against RSV [41,42]. The disadvantages include elevated expenses and the necessity for equipment upkeep and worker training [44].

The cord RSV IgG antibody levels correlated with disease severity in the first 6 months.

Serology is limited as a diagnostic tool due to seroconversion occurring over two weeks, the inability to identify virus-specific antibodies in newborns with RSV infections, and the presence of maternal antibodies. The direct fluorescence antibody test yields results within 2–3 h, exhibiting a sensitivity and specificity of approximately 95%, albeit it requires specialized knowledge. Diagnostic serology is unhelpful in assessing and managing RSV infection due to the presence of maternal antibodies in infants and the consistent levels of RSV-specific antibodies in older children [45].

Viral cell culture is the conventional method for diagnosing RSV, albeit results need approximately 3 to 7 days. Rapid cell culture (shell-vial) produces results within 48 h, in contrast to traditional cell culture methods.

The chest X-ray reveals hyperinflation, diaphragm flattening, infiltrations, atelectasis, and pronounced parabronchial shadows, allowing for the exclusion of alternative diagnoses.

Nasal lavage, nasopharyngeal swab, and throat swab are viable sampling procedures; however, bronchoalveolar lavage and tracheal aspirate sampling are necessary for intubated patients. These samples exhibit enhanced sensitivity for virus detection [46].

### 1.5. Differential Diagnosis

- Asthma;
- Bronchiolitis;
- Influenza;
- Croup;
- Bronchitis;
- Pneumonia.

### 1.6. Treatment

Supportive treatment and antiviral medication are predominantly the main elements of therapeutic intervention for RSV infection. Monitoring the patient’s clinical status, administering fluids and paracetamol, and providing respiratory aid as necessary are all elements of supportive care. Patients exhibiting severe respiratory symptoms or apnea due to RSV may necessitate mechanical ventilation or alternative kinds of treatment. Ribavirin is the most utilized antiviral medication.

Ribavirin is a synthetic nucleoside analog with significant in vitro efficacy against RSV and is sanctioned by the U.S. Food and Drug Administration (FDA). Nonetheless, it is not regularly advised for infants and children due to the lack of established efficacy [47,48]. It is costly and should be administered early in the treatment to be effective, considering concerns over occupational exposure [47]. It is contraindicated in pregnant women due to the danger of teratogenic effects. Adverse effects encompass hemolytic anemia, leukopenia, cough, bronchospasm, rash, and conjunctival irritation [49,50,51,52]. It is linked to bronchoconstriction and necessitates vigilance in individuals with asthma or chronic obstructive pulmonary disease [49].

Randomized controlled trials evaluating ribavirin versus placebo in pediatric patients with RSV LRTI yield inconclusive results, with some studies indicating reduced illness severity, shorter durations of mechanical ventilation, oxygen therapy, and hospitalization, as well as diminished viral shedding, while others report no significant benefit.

The American Academy of Pediatrics advises against the routine use of ribavirin due to prolonged aerosol exposure, hospitalization risks, potential for toxicity (bone marrow suppression, carcinogenic effects), teratogenicity, and significant expense [53,54]. Immunocompromised individuals with severe RSV infection should be the only ones prescribed ribavirin, and their usage should only be authorized by an infectious disease specialist.

Intravenous immune globulin exhibiting elevated neutralizing activity against RSV (RSV-IVIG) is a hyperimmune polyclonal immunoglobulin derived from donors possessing high levels of RSV-neutralizing antibodies. It has five times better efficacy in neutralizing RSV than IVIG. It received FDA approval in 1996 for the reduction of hospitalizations in high-risk newborns. It is no longer accessible due to RCTs indicating no advantage [55].

## 2. Patients and Method

From August 2022 to March 2024, 1903 babies in Romania eligible for immunoprophylaxis with Palivizumab were included in a non-interventional, observational, retrospective study conducted in grade 2 and 3 maternity hospitals. These patients were enrolled in the national immunoprophylaxis program. All patients received Palivizumab intramuscular at a dose of 15 mg/kg.

The analysis included the aspects of the perinatal period, the clinical status of the baby at the time of inclusion, the immunization schedule, adherence to the Palivizumab doses, and the adherence of the medical specialists. All data were collected in a national report and analyzed in Microsoft Excel.

The inclusion criteria comprised premature infants born before 34 weeks, infants with congenital heart disease, and infants with bronchopulmonary dysplasia. The exclusion criterion was deceased patients who could no longer be contacted. Follow-up was conducted by telephone interview. All relatives were contacted by telephone before the delivery of each dose to confirm the execution of the immunoprophylaxis protocol. All legal guardians signed a consent form prior to the administration. The study has the ethics committee approval no. 9483/02.10.2024.

## 3. Results

The program is structured to provide services variably according to the birth month. The delivery of program services to caregivers varies based on the timing of birth relative to the onset of the cold season. Premature infants born during the warm season from April to September do not receive Palivizumab administration but are enrolled in the program. Infants born during the cold season from October to March are enrolled in the program and receive the first dose in the first days of life. All enrollments receive a welcome call, accompanied by an educational email, followed by additional educational and appointment calls, as well as one to four educational emails and one to four reminder SMS messages. In Romania, the seasonality of RSV infection exists between October and March, and immunoprophylaxis is administered according to the month of birth.

**Figure 1:**
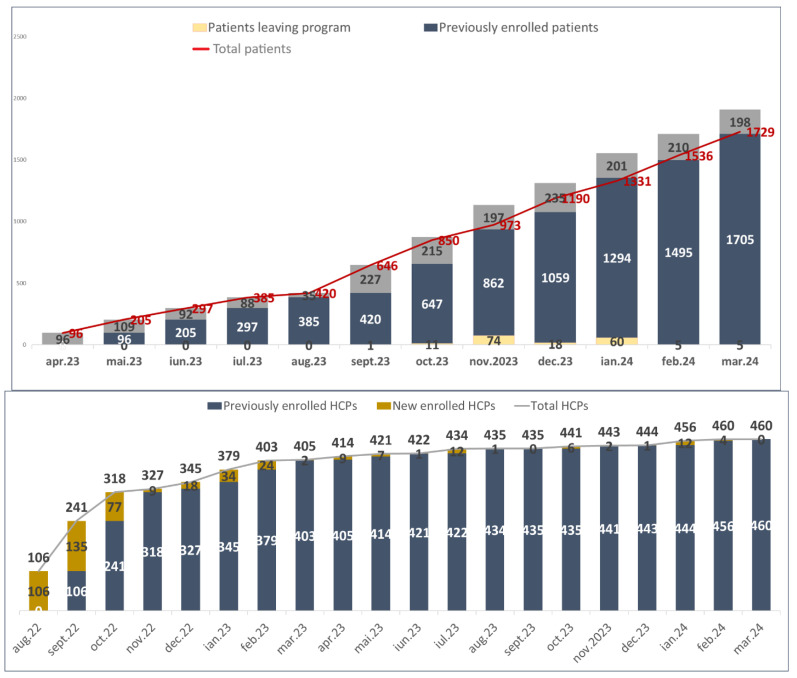
Patients and specialists (HCPs) enrolled. In the red line is the total number of patients, and in the gray portions are the newly enrolled patients. In the gray line are the specialists enrolled, and in the yellow portions are the newly enrolled physicians.

Since the beginning of the program in August 2022, a total of 1903 patients and 233 specialists have been registered for participation (Figure 1).

### 3.1. Patients with 5 Possible Administrations—Estimated Adherence

A total of 1059 patients were enrolled between April and November 2023 and could administrate Palivizumab five times until March 2024.

**Figure 2:**
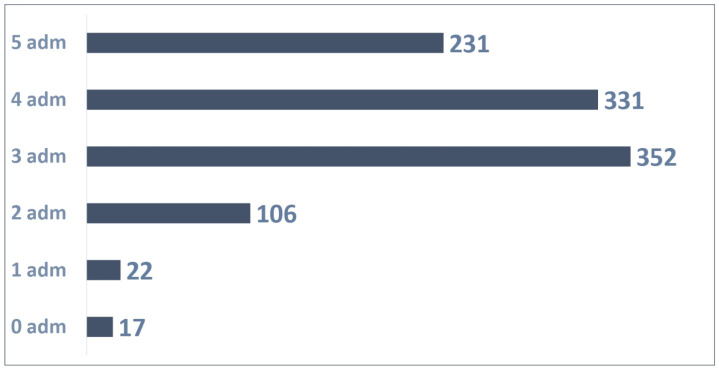
Patients with 5 possible administrations.

The distribution of 1059 patients, as presented in Figure 2, based on the number of administrations was as follows:

- A total of 17 patients were not administrated Palivizumab;
- A total of 22 patients received one Palivizumab administration;
- A total of 106 patients received two Palivizumab administrations;
- A total of 352 patients received three Palivizumab administrations;
- A total of 331 patients received four Palivizumab administrations;
- A total of 231 patients received five Palivizumab administrations.

**Figure 3:**
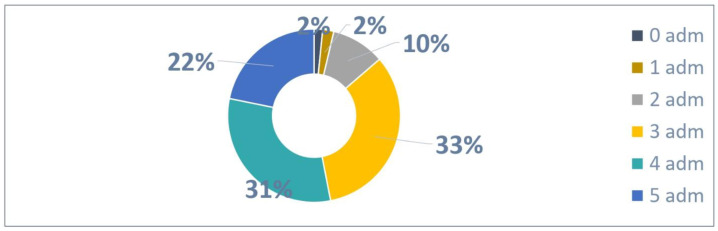
Adherence to 5 doses in patients.

A total of 3769 administrations were reported out of 5292 possible (71% adherence rate) as detailed in Figure 3.

An average of 3.56 administrations per patient were performed out of 5 possible.

### 3.2. Patients with 4 Possible Administrations—Estimated Adherence

A total of 235 patients were enrolled in Dec 2023 and could administrate Palivizumab four times until March 2024.

**Figure 4:**
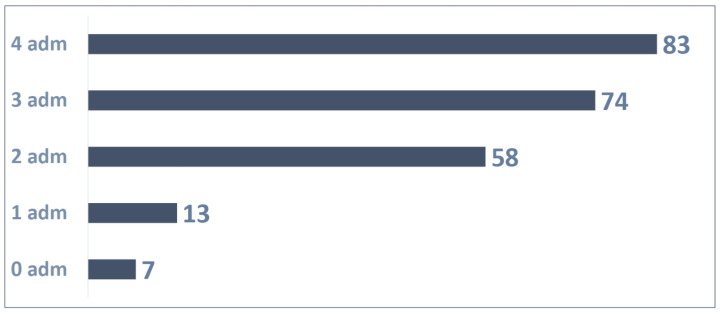
Patients with 4 possible administrations.

The distribution of the 235 patients (Figure 4) based on the number of administrations was as follows:

- Seven patients were not administrated Palivizumab;
- A total of 13 patients received one Palivizumab administration;
- A total of 58 patients received two Palivizumab administrations;
- A total of 74 patients received three Palivizumab administrations;
- A total of 83 patients received four Palivizumab administrations.

**Figure 5:**
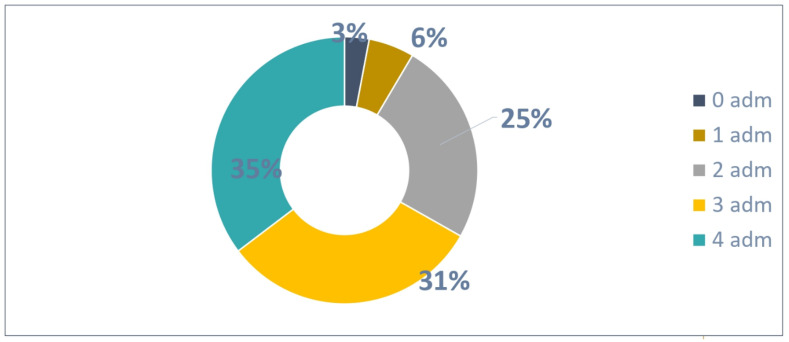
Adherence to 4 doses in patients.

A total of 683 administrations were reported out of 940 possible (73% adherence rate) as shown in Figure 5.

An average of 2.91 administrations per patient were performed out of 4 possible.

### 3.3. Patients with 3 Possible Administrations—Estimated Adherence

A total of 201 patients were enrolled in January 2024 and could administrate Palivizumab three times until March 2024

**Figure 6:**
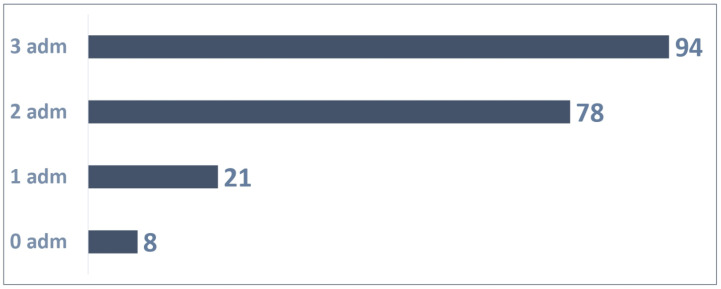
Patients with 3 possible administrations.

The distribution of the 201 patients (Figure 6) based on the number of administrations was as follows:

- Eight patients were not administered Palivizumab;
- A total of 21 patients received one Palivizumab administration;
- A total of 78 patients received two Palivizumab administrations;
- A total of 94 patients received three Palivizumab administrations.

**Figure 7:**
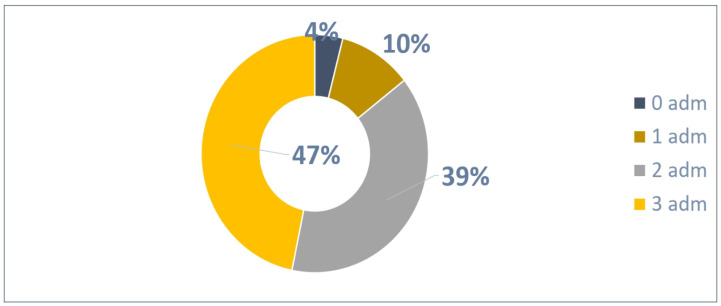
Adherence to 3 doses in patients.

A total of 459 administrations were reported out of 603 possible (76% adherence rate) as illustrated in Figure 7.

An average of 2.28 administrations per patient were performed out of 3 possible.

### 3.4. Patients with 2 Possible Administrations—Estimated Adherence

A total of 210 patients were enrolled in February 2024 and could administrate Palivizumab two times until March 2024.

**Figure 8:**
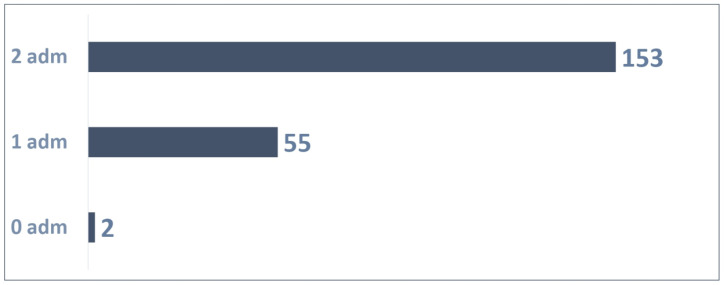
Patients with 2 possible administrations.

The distribution of 210 patients (Figure 8) based on the number of administrations was as follows:

- Two patients were not administrated Palivizumab;
- A total of 55 patients received one Palivizumab administration;
- A total of 153 patients received two Palivizumab administrations.

**Figure 9:**
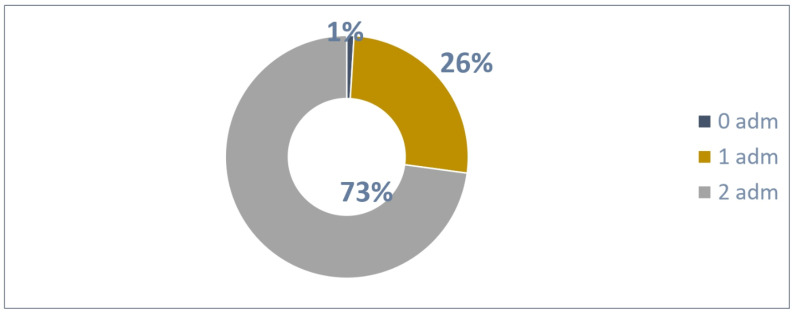
Adherence to 2 doses in patients.

A total of 361 administrations were reported out of 420 possible (86% adherence rate) according to Figure 9.

An average of 1.72 administrations per patient were performed out of 2 possible.

### 3.5. Patients with 1 Possible Administration—Estimated Adherence

A total of 198 patients were enrolled in March 2024 and could administrate Palivizumab one time until the cold season ends.

**Figure 10:**
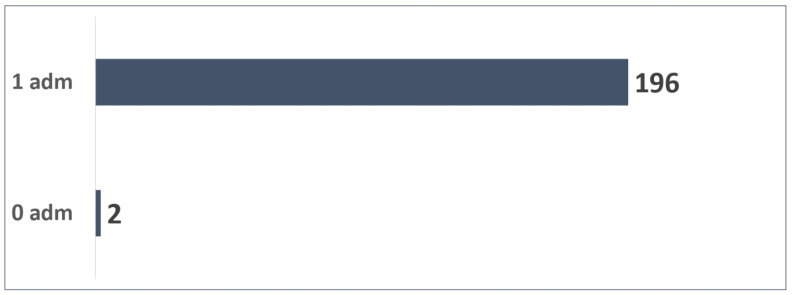
Patients with 1 possible administration.

The distribution of the 198 patients (Figure 10) based on the number of administrations was as follows:

- Two patients were not administrated Palivizumab;
- A total of 196 patients received one Palivizumab administration.

**Figure 11:**
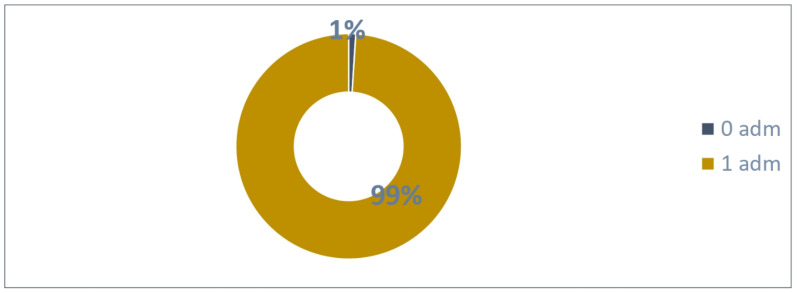
Adherence to 1 dose in patients.

A total of 196 administrations were reported (99% adherence rate) as in Figure 11.

An average of 0.99 administrations per patient were performed.

## 4. Discussion

Following the 2014 AAP guidelines on Palivizumab prophylaxis for RSV infections, prophylaxis was restricted to preterm infants born at 29 0/7 weeks of gestation without underlying chronic lung disease or congenital heart disease, while the threshold for infants with chronic lung disease was set at 32 0/7 weeks of gestation. Prophylaxis will be administered in the second year to preterm infants with chronic lung illness who have undergone oxygen therapy for a minimum of 28 days and have received steroids, bronchodilators, or oxygen from the onset of the second RSV season until six months prior. It was determined that infants under 12 months with hemodynamically severe congenital heart disease should receive Palivizumab prophylaxis; however, this prophylaxis should not be extended beyond the second year for these patients. Patients undergoing treatment for congestive heart disease, as well as those with cyanotic heart disease necessitating cardiac surgery and pulmonary hypertension, would significantly benefit from prophylaxis. In contrast to the 2012 guidelines, it has been reported that prophylaxis should be terminated in infants hospitalized due to RSV while undergoing RSV prophylaxis [56].

The prevention of RSV infection in children at risk (born at 35 weeks of gestation or less and less than 6 months of age at the onset of the RSV season) is implemented through a Patient Support Program combining educational and pro-adherence interventions at the level of neonatologists from hospital maternity and pediatricians involved in prescription issuing and caregivers. Prophylaxis varies by country, as does metabolic testing, which encompasses different disorders [57].

RSV immunization during gestation, which involves the acquisition of maternal antibodies transmitted transplacentally to avert problems like preterm birth and early rupture of membranes, is another short-term preventive strategy [58].

Research on RSV immunoprophylaxis has examined many monoclonal antibodies (mAbs), finding that Nirsevimab, Motavizumab, and Palivizumab showed efficacy.

Nirsevimab is a monoclonal antibody characterized by an extended half-life and significant neutralizing efficacy. A multi-center, placebo-controlled, randomized controlled trial with healthy infants born at ≥29 weeks gestation showed that a single injection of Nirsevimab effectively prevented RSV lower respiratory tract infections and hospitalization for 150 days [59,60]. It has recently received approval for clinical application [61]. Due to the necessity of a single administration, adherence to therapy would likely be enhanced; nonetheless, it is unavailable in Romania, similar to Motavizumab.

Palivizumab is the most widely used humanized IgG1 monoclonal antibody specific to RSV immunoprophylaxis. It obstructs viral replication by impeding its attachment to the respiratory epithelium [55]. It is generated through recombinant DNA technology and received licensure in 1998 for the prophylaxis of severe RSV lower respiratory tract illness in high-risk pediatric populations. Administered intramuscularly at a dosage of 15 mg/kg monthly for a total of five treatments to sustain a serum concentration exceeding 40 μg/mL in preterm infants with bronchopulmonary dysplasia (BPD). Monthly dosing is recommended due to an average half-life of roughly 20 days [62].

It has been demonstrated that the immune response and the production of antibodies are dependent on the dose, so an insufficient dose does not provide immunity to newborns [63,64]. Adverse responses are exhibited by fever, rash, and antibody production. A meta-analysis comparing Palivizumab prophylaxis to placebo in preterm infants ≤35 weeks’ gestation with bronchopulmonary dysplasia and congenital heart disease showed that Palivizumab decreased RSV hospitalizations without a rise in side events. Palivizumab prophylaxis is indicated for infants with bronchopulmonary dysplasia (BPD) under 1 year of age at the onset of the RSV season [50,65] or for those aged 12 to 23 months who necessitate medical therapy for BPD [66].

An administration of Palivizumab is possible during pregnancy. During pregnancy, the amount of IgG antibodies that are administered to the fetus gradually declines throughout the pregnancy, reaching its lowest point between the ages of two and three months pregnant.

When dealing with RSV-infected neonates, it is important to adhere to standard measures, including proper hand cleanliness, nursing, and contact isolation. When it comes to the prevention of RSV, the Centers for Disease Control and Prevention (CDC) suggests taking both basic precautions and contact precautions. Healthcare providers need to take precautions such as cleaning their hands, using gloves, wearing a surgical mask, wearing eye protection, and wearing disposable gowns [67,68].

Infected patients must be isolated using standard and contact precautions in private rooms or grouped with other RSV-infected patients in designated rooms [67,69].

The positive and negative variables of adherence to immunoprophylaxis with Palivizumab were investigated in a comprehensive review that was published in 2010. The review included twelve different bodies of research. The perceptions of the parents were found to be the most significant predictor of individuals’ adherence to immunoprophylaxis, according to the findings of the study [70].

Among the 78% of children who finished all of their doses, 67% of parents believed that Palivizumab is the most effective defense against severe RSV infections. This contrasts with the 48% of parents who were in the group that did not comply with the treatment [71].

The percentage of patients that complete their full sequence of doses decreases along with the number of doses (99% for one dose, 73% for two doses, 47% for three doses, 35% for four doses, and 22% for five doses) because of insufficient parental compliance (owing to a lack of faith in the efficacy of Palivizumab), restricted availability to therapy, and language difficulties were the primary negative predictive factors for adherence to immunoprophylaxis with Palivizumab. Other factors were parental smoking, insufficient medical personnel in underprivileged settings, and the lack of a national immunoprophylaxis program. Follow-up telephone interviews determined the factors contributing to reduced adherence.

The researchers Chow et al., reported in 2017 that there was a large static increase in compliance among programs that included basic telephone reminders to parents or caregivers, multidisciplinary interventions that included counseling, reminder calls the day before appointments, and nurses reviewing charts [72,73].

In Romania, 176 out of 1903 patients completed the program between September 2023 and March 2024, and the main barriers to immunoprophylaxis administration were as follows:

- A total of 29%: Do not want to administrate the treatment anymore;
- A total of 28%: HCP advice against the treatment;
- A total of 18%: Difficult access to treatment;
- A total of 15%: Family against the treatment;
- A total of 10%: Others.

The limitations of the study were that it was an observational study without a comparative group, administrative factors like doctors who refused to administer Palivizumab without scientific argument, and newborns born in centers that were not part of the program.

## 5. Conclusions

The program continues to be very well received by both doctors and caregivers, showing it to be a highly helpful resource for educating parents and supporting treatment administrations by medical prescription. This was accomplished by building upon the favorable outcomes that were achieved in the first year of intervention.

To achieve a very high adherence rate of 73%, the program assisted caregivers so that they could overcome the challenges and carry out treatment administrations following the recommendations of the doctor.

The effectiveness of the immunization program is chiefly attributable to the personnel engaged in conducting telephone interviews, reminding patients of Palivizumab injection dates, establishing contacts among local family physicians, and coordinating appointments. The decline in adherence to dosage administration is also attributable to the insufficient availability of medical workers in certain regions of the country.

Concerning the administration of Palivizumab, significant obstacles continue to exist, the most significant of which is being generated by the present anti-vaccine and bioecologist trends. It is essential to provide caregivers with accurate information that is disseminated through program activities to educate and assist them in their efforts to provide the necessary protection for their premature children. Further studies should be performed to identify populations prone to low adherence to immunoprophylaxis administration with Palivizumab.

## Data Availability

Data are provided by AstraZeneca Romania through Totem Communication.
